# Total laboratory automation-based monitoring processes: setup and validation of an integrated internal quality control panel

**DOI:** 10.3389/fcimb.2026.1771552

**Published:** 2026-05-06

**Authors:** Adrien Fischer, Abdessalam Cherkaoui, Didier Schorderet, Julie Wagner, Jacques Schrenzel

**Affiliations:** Bacteriology Laboratory, Department of Diagnostics, Geneva University Hospitals, Geneva, Switzerland

**Keywords:** quality costs, diagnostic laboratory, internal quality control, microbiology, reference strains, total laboratory automation (TLA)

## Abstract

**Background:**

Total laboratory automation (TLA) is now widely implemented in diagnostic microbiology laboratories. However, a coherent and standardized quality surveillance procedure for all the automated culture-based processes is currently lacking.

**Objectives:**

We set up and implemented an internal quality control (IQC) panel to assess the automated culture-based processes and workflows.

**Methods:**

A subset of reference strains was applied to monitor liquid and solid culture media used in all culture-based processes on the TLA. This integrated IQC panel monitors staining, automated plate streaking, incubation and digital imaging, artificial intelligence (AI)-assisted plate reading, identification by MALDI-ToF-MS, fully automated antimicrobial disc diffusion susceptibility testing, minimum inhibitory concentration (MIC) determination by broth microdilution and E-test strips, and finally, detection of the defined antimicrobial resistance genes by molecular assays.

**Results:**

During 6 months of implementation of this new routine IQC approach, no errors were detected regarding all the culture-based and antimicrobial susceptibility testing (AST) processes, including antimicrobial resistance gene detection, with the exception of one major error related to a MIC misreading for imipenem.

**Conclusions:**

This IQC approach ensures the traceability and control of the analytical phase of an automated laboratory. Annual costs for these integrated IQC panels amounted to CHF 17,886.-, of which 53% were related to personal time costs. The latter could be improved by further software development for sample prescription, data collection, and reporting.

## Introduction

Nowadays, total laboratory automation (TLA) coupled to artificial intelligence in clinical microbiology laboratory implies a fundamental streamline of the phenotypic analytical processes (Cherkaoui et al., 2025b; Graham et al., 2025; Van et al., 2019). Improving diagnostic accuracy, efficiency, and productivity are the key goals that can be achieved (Bailey et al., 2019; Cherkaoui et al., 2023, 2019; Dauwalder et al., 2016). The development of integrated strategies to minimize human errors, enhance quality control, and provide sample tracking remains a pivotal quality assurance objective for a successful implementation of the TLA.

Full automation requires a variety of devices that are rarely produced by the same manufacturer (Dauwalder et al., 2016). While IVD-R certification was documented for many of them, a coherent and standardized quality surveillance procedure when these devices operate together is still difficult to implement, and no specific recommendations exist. The same observation can be made concerning the reagents.

The purpose of the present study was to set up and validate a comprehensive internal quality control (IQC) procedure to ensure accurate surveillance of all phenotypic analytical processes on the TLA, and to systematically monitor the output results.

## Material and methods

### Strains

Defined IQC strains (**Table 1**) are microorganism reference strains available from the ATCC (as indicated by their ATCC number) or confirmed by the national reference center for early detection and monitoring of antibiotic resistance (NARA, Fribourg, Switzerland) as antimicrobial resistance gene producers.

**Table 1:** Procedures used for monitoring automated cultures, including negative swabs.

IQC strains and controls	Seeding protocol^a^	Staining	MALDI-ToF-MS ID (monthly)	Radian AST (weekly)	Sensititre AST (monthly)	E-test AST (monthly)	MDx panels (monthly)
*Staphylococcus aureus* ATCC 29213	1	Gram	x	x		DAP/TEIC/VANC	
*Pseudomonas aeruginosa* ATCC 10145	1 and 3	AO	x	x			
*Escherichia coli* ATCC 25922	1 and 3	Gram	x	x	MDR panel^b^	AMC/CIP/MERO	
*Enterococcus faecalis* ATCC 29212	1 and 3		x	x			
*Haemophilus influenzae* ATCC 10211	1		x			AMP/CEFTR	
*Streptococcus agalactiae* ATCC 12386	4		x				
*Staphylococcus aureus* MRSA USA300 JE2 (Diep et al., 2006)	2		x				
*Enterococcus faecium* VRE ATCC 700221	2		x				
*Escherichia coli* -9 ESBL	2		x				Amplex Carba^c^
*Klebsiella pneumoniae* -10 Oxa48	2		x				Amplex Carba^c^
*Pseudomonas aeruginosa* -11 VIM	2		x		MDR panel^b^		Amplex Carba^c^
*Acinetobacter baumanii* -12 Oxa23	2		x				Amplex Carba^c^
*Pichia kudriavzevii* (previously: *Candida krusei*) ATCC 6258	1	Calcofluor/Fungi-Fluor^d^	x		Fungi panel^e^		
*Bacteroides fragilis* ATCC 25285	5		x		Anaerobic panel^f^		
E-swab 1 (negative control)	1		(x)				
E-swab 2 (negative control)	2		(x)				
E-swab 3 (negative control)	3		(x)				
E-swab 4 (negative control)	4		(x)				

AO, Acridine Orange; AMC, amoxicillin–clavulanate; AMP, ampicillin; CEFTR, ceftriaxone; CIP, ciprofloxacin; DAP, daptomycin; MERO, meropenem; TEIC, teicoplanin; VANC, vancomycin.

^a^ Seeding protocols are detailed in **Table 2**.

^b^ Microdilution AST for multidrug-resistant strains contained the following antibiotics: amikacin, aztreonam, cefepime, ceftazidime/avibactam, ceftolozane/tazobactam, colistin, eravacycline, fosfomycin (+glucose-6-phosphate), imipenem/relebactam, imipenem, meropenem/vaborbactam, meropenem, piperacillin/tazobactam, tigecycline, and tobramycin.

^c^ Resistance gene screening by molecular assays contained the following targets: CTX M 1 group, CTX M 9 group, KPC, NDM, Oxa23, Oxa40, Oxa48, Oxa58, Oxa181, and VIM. (x): If growth = contamination.

^d^ Calcofluor and Fungi-Fluor refer to the specific staining methods for molds and yeasts.

^e^ Microdilution AST for molds and yeasts contained the following antifungals: anidulafungin, amphotericin B, micafungin, caspofungin, 5-flucytosine, posaconazole, voriconazole, itraconazole, and fluconazole.

^f^ Microdilution AST for anaerobic bacteria contained the following antibiotics: amoxicillin, amoxicillin/clavulanic acid, cefoxitin, chloramphenicol, clindamycin, erythromycin, imipenem, metronidazole, penicillin, piperacillin/tazobactam, tetracycline, and vancomycin.

### Staining protocols

The staining procedures are detailed in **Table 1**. All glass slides were prepared by the WASP (COPAN, Brescia, Italy) and stained for Gram (Previ^®^ Color Gram, 053815-02) on the PreviColor (bioMérieux, Marcy-L’Etoile, France) or manually stained for Acridine Orange (AO, BBL™, BD, Heidelberg, Germany, 212537). Calcofluor (BactiDrop™, Thermo Fisher Scientific, Landsmeer, The Netherlands, 21507) and Fungi-Fluor (Polysciences, Warrington, USA) staining were performed manually and used for the detection of yeasts and molds.

### Culture media

All the routine culture media used in our lab were included in this integrated IQC procedure. The culture media manufactured by bioMérieux (bioMérieux SA, Marcy-L’Etoile, France) were as follows: COS (blood agar + 5% sheep blood), MCK (Mac Conkey agar), CNA (Columbia agar), PVX (chocolate agar + PolyViteX™) (lysed red blood cells), MHE (Mueller–Hinton E agar) for antimicrobial susceptibility testing (AST) by disc diffusion and E-tests, MHF (Mueller–Hinton agar with 5% horse blood + 20 mg/L of β-NAD) for AST by disc diffusion and E-tests for fastidious organisms, SA-ID (CHROMID^®^ *Staphylococcus aureus* Elite - chromogenic media) for the screening of *S. aureus*, MRSA-ID (CHROMID^®^ MRSA agar - chromogenic media) for the screening of methicillin-resistant *S. aureus*, VRE-ID (CHROMID^®^ VRE agar - chromogenic media) for the screening of vancomycin-resistant enterococci, ESBL-ID (CHROMID^®^ ESBL agar - chromogenic media) for the screening of Enterobacterales with an extended spectrum beta-lactamase, OXA-ID (CHROMID^®^ OXA-48 agar - chromogenic media) for the screening of carbapenemase-producing Enterobacterales, CPSE (CHROMID^®^ CPS^®^ Elite - chromogenic media) for the screening of common urinary tract pathogens, STRB (CHROMID^®^ Strepto B agar) for the screening of *Streptococcus agalactiae*, and SCS (Schaedler agar + 5% sheep blood) for anaerobic cultures. The culture medium from Axon Lab (Axon Lab AG, Switzerland) was Acin-ID (CHROMagar™ *Acinetobacter* MDR agar) for the screening of *Acinetobacter* spp. The culture medium from COPAN (COPAN, Brescia, Italy) was BHI (Brain Heart Infusion broth). The culture medium from BD (Becton Dickinson GmbH, Heidelberg, Germany) was CAC (BBL™ CHROMagar™ Candida Medium - Yeast Extract Glucose Chloramphenicol agar) for growing yeasts. One homemade medium was used: BHI-GC (Brain Heart Infusion agar with gentamycin and chloramphenicol) for growing molds.

### Antimicrobial susceptibility testing

AST by disc diffusion using the Radian system (COPAN, Brescia, Italy) was performed as previously described (Cherkaoui et al., 2021). Automated disc diffusion reading and preliminary interpretation were performed by the Radian expert system. All antibiogram results were reviewed by a technologist before separate validation by a microbiologist.

Minimum inhibitory concentration (MIC) determinations were performed using the microdilution plates Sensititre™ (Thermo Fisher Scientific, Landsmeer, The Netherlands) and E-tests (bioMérieux SA, Marcy-L’Etoile, France). Three different Sensititre™ plates were used: the MDR panel (Sensititre™ EUMDRXXF), the anaerobic panel (Sensititre™ ANAERO3), and the fungi panel (Sensititre™ YEASTONE™ YO10). MIC reading was performed by a technologist, while interpretation and validation were done by a microbiologist.

Disc diffusion diameters and MIC were interpreted following EUCAST recommendations (EUCAST v15; https://www.eucast.org/ast_of_bacteria/quality_control).

### Minimum inhibitory concentration range and targets

Whenever a strain used in our IQC panel was not referenced in EUCAST QC tables, the MIC range was determined as proposed by EUCAST by defining one dilution above and one dilution under the target value. MIC target values were determined as the median value from five independent AST determinations. This was applied to the E-tests for *Haemophilus influenzae*, the broth dilution Sensititre™ for *Pseudomonas aeruginosa* VIM (not an ATCC strain), and some antibiotic drugs from the anaerobic Sensititre™ broth dilution plate for *Bacteroides fragilis* (amoxicillin, cefoxitin, chloramphenicol, erythromycin, moxifloxacin, penicillin, piperacillin, tetracycline, and vancomycin).

### Molecular assays

Antimicrobial resistance gene detection was performed using the Eazyplex^®^ system (Eazyplex^®^ SuperBug CRE, Amplex Diagnostics GmbH, Germany).

### Costs

Costs were estimated based on the time dedicated to IQC determination and reporting, as well as on the number of kits and consumables used for IQC during the year 2025. The number of MALDI-ToF-MS spots referred only to those ordered by the WebApp using Colibri^®^.

The technologists’ time recorded covers time management of the reference strains, time IQC preparation, analyzing tasks, and results collection.

Dedicated and routine technologist times are allocated separately for IQC (IQC load) but shared for routine automated culture analysis (routine load).

## Results

**Figure 1:**
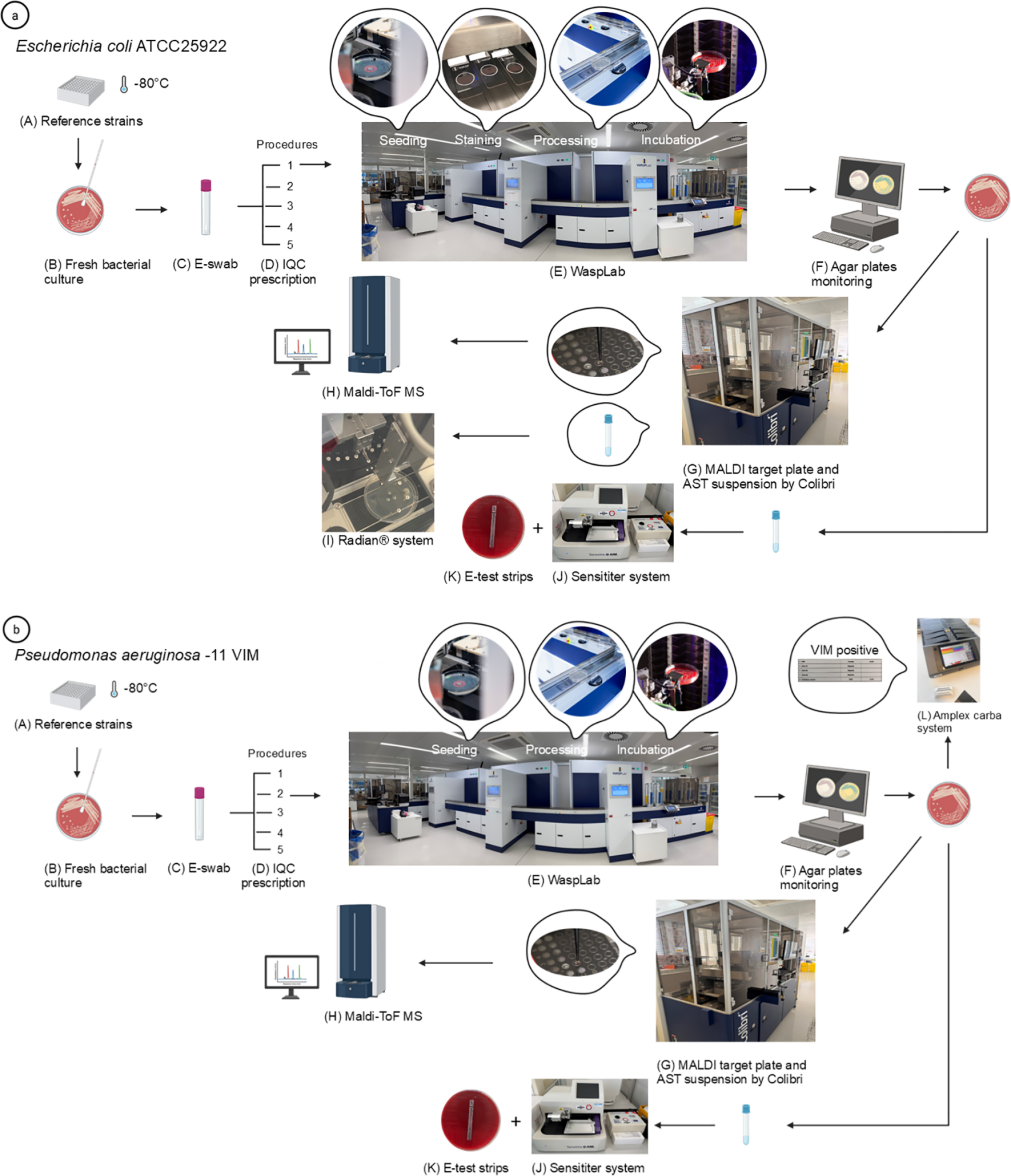
Illustration of the steps monitored by the integrated IQC panel. Two reference strains, *Escherichia coli* ATCC25922 **(a)** and *Pseudomonas aeruginosa* -11 VIM **(b)**, are used to depict the steps monitored by this IQC panel. (A) Reference strains are aliquoted for single use and stored at −80 °C. (B) Aliquots are plated to obtain fresh bacterial cultures and (C) sampled on an E-swab. (D) Prior to seeding on the automatic workflow, a manual prescription is generated to process the sample. Strain-specific seeding protocols and automated analyses are then performed by the WASPLab. **(a)**: (E) For *E. coli* ATCC25922, WASPLab performs seeding on COS, CNA, MCK, PVX, BHI-GC, and CAC agar plates, a BHI broth, and glass smears for offline staining (Gram – AO – Calcofluor – Fungi-Fluor). Agar plates are loaded into the WASPLab incubators, and the BHI broth is loaded into stand-alone incubators. Following specified incubation times, plate imaging is carried out, and (F) bacterial growth on each agar plate is monitored by a lab technologist to define colonies to be picked up. The automated Colibri® prepares a MALDI target plate (G) for bacterial identification using MALDI-ToF-MS (H) and a calibrated AST suspension for AST determination using the automated Radian^®^ system (I). MIC determinations are performed whenever defined using broth dilution with the Sensititre system (J) and E-test strips (K). **(b)** (E) For *P. aeruginosa* -11VIM, WASPLab performs seeding on SA-ID, MRSA-ID, VRE-ID, Acin-ID, ESBL-ID, and OXA-ID agar plates. Agar plates are loaded into the WASPLab incubators. Following specified incubation times, plate imaging is carried out, and (F) bacterial growth on each agar plate is monitored by a lab technologist to define colonies to be picked up. The automated Colibri® prepares a MALDI target plate (G) for bacterial identification using MALDI-ToF-MS (H). MIC determination is performed by broth dilution using the Sensititre system (J) and E-test strips (K). Loop amplification using the Amplex Carba system (MDx panel) detects the presence of the antibiotic-resistant gene VIM (L). This figure was created in BioRender (www.biorender.com).

**Figure 1** and **Table 1** depict microorganism reference strains that were applied to monitor staining, automated plate streaking, incubation and digital imaging, artificial intelligence (AI)-assisted plate reading, identification by MALDI-ToF-MS, fully automated antimicrobial disc diffusion susceptibility testing, MIC determination by broth microdilution and E-test strips, and finally, detection of the defined antimicrobial resistance genes by molecular assays.

Our objective was to set up a user-friendly, robust, and practical IQC panel. Due to the large number of culture media used in our routine and the requirements related to WASP capacity, we generated a series of seeding protocols based on the media plates (**Table 2**).

**Table 2:** Detailed seeding protocols, including agar plates and incubation times.

Seeding protocol	Protocol name	Agar plates	Incubation time (h)
1	Non-chromogenic agar	COS	20
		CNA	20
		MCK	20
		PVX	20
		BHI-GC	72
		CAC	20
		BHI	20
2	Chromogenic agar	SA-ID	20
		MRSA-ID	20
		VRE-ID	20
		Acin-ID	20
		ESBL-ID	20
		OXA-ID	20
3	CPS	CPSE	20
4	STRB	STRB	20
5	Ana	COS	20
		MCK	20
		CNA	20
		PVX	20
		SCS	48

Non-chromogenic agar: agar plates for non-selective growth or specific Gram type growth (Gram negative vs. Gram positive); chromogenic agar: agar plates for selective growth based on resistance and bacterial colony color; CPS: procedure for urine culture; STRB: procedure for *Streptococcus agalactiae* screening; Ana: refers to the anaerobic growth procedure, but only SCS agar is incubated in anaerobiosis. COS: blood agar, MCK: MacConkey agar, CNA: Columbia agar, PVX: chocolate agar (lysed red blood cells), BHI-GC: Brain Heart Infusion agar with gentamycin and chloramphenicol, CAC: yeast extract glucose chloramphenicol agar, BHI: Brain Heart Infusion broth, SA-ID: chromogenic media for the screening of *S. aureus*, MRSA-ID: chromogenic media for the screening of methicillin-resistant *S. aureus*, VRE-ID: chromogenic media for the screening of vancomycin-resistant enterococci, Acin-ID: chromogenic media for the detection of *Acinetobacter* spp. and MDR *Acinetobacter*, ESBL-ID: chromogenic media for the screening of Enterobacterales with extended spectrum beta-lactamase, OXA-ID: chromogenic media for the screening of carbapenemase-producing Enterobacterales, CPSE: chromogenic media for the screening of common urinary tract pathogens, STRB: chromogenic media for the screening of *Streptococcus agalactiae*, SCS: anaerobic blood agar.

Culture media were grouped to mimic routine workflow, and reference strains were selected for positive and negative growth in each seeding protocol. We defined a total of five different seeding protocols (**Table 2**) in our routine. Seeding protocol 1 included all non-chromogenic agar plates to test strains like *Escherichia coli* ATCC25922 (**Figure 1a**). Chromogenic agar plates were assigned to seeding protocol 2. Chromogenic agar plates were mostly used for routine hospital screening for multidrug-resistant bacteria (e.g., VRE, carbapenemase-producing Enterobacterales, MRSA, or multiresistant *Acinetobacter baumanii* strains). Strains like *P. aeruginosa* -11 VIM were seeded using protocol 2 to assess the bacterial growth on OXA and ESBL plates and the absence of growth on the other plates (**Figure 1b**). Seeding protocols 3 and 4 used one plate each, for urine (CPSE) and *S. agalactiae* screening (STRB), respectively. Seeding protocol 5 was dedicated to the anaerobic culture (Ana) using a separate offline anaerobic incubator (Scholzen, Microbiology Systems AG, Switzerland). Incubation times were defined based on IQC requirements. In routine, we inoculated samples with variable (often low) bacterial loads. For this integrated IQC, we started from freshly isolated strain dilutions, and very high bacterial (or yeast) loads were seeded on culture media, consistent with our aim to monitor potential contaminations (e.g., improper decontamination of the automated system or agar contamination) and to ensure correct species identification across the whole procedure. For these reasons, we selected 20 h of incubation for most agar plates, 48 h for anaerobic cultures (like in our routine workflow), and 72 h for the BHI-GC agar plate used for recovering yeasts and molds.

After seeding, the WASP system automatically prepares smears for microscopic staining. Slide preparation was exclusively addressed by seeding protocol 1, where a laboratory technologist performs Gram, Acridine Orange, Calcofluor, or Fungi-Fluor staining on the relevant strains (**Table 1**).

After incubation, a single determination by MALDI-ToF-MS was deemed sufficient whenever bacteria growing on the media plates appeared homogeneous and consistent with the identification. Whenever another morphology was detected on any plate, or if MALDI-ToF-MS identification yielded an incorrect result, an error flag was reported (**Table 3**), and additional investigations were applied to analyze the case.

**Table 3:** Results interpretation for the culture on the various agar plates.

IQC	Gram (+/−) and shape	AO shape	Calcofluor (+/−)	Fungi-Fluor (+/−)	Growth/culture media	MALDI-ToF-MS ID	Radian AST	Sensititre AST	E-test AST	MDx							
*E. coli* ATCC 25922	GNR	Rod	ND	ND	V	V	V	V	V	ND							
*P. aeruginosa* -11 VIM	GNR	Rod	ND	ND	V	V	V	V	V	VIM							

Growth/color (+/−)	CPSE	STRB	COS	CNA	MCK	PVX	SCS	BHI-GC	CAC	BHI	SA-ID	MRSA-ID	VRE-ID	Acin-ID	ESBL-ID	OXA-ID	Final growth validation
*E. coli* ATCC 25922	+/+	ND/ND	+/ND	+/ND	+/+	+/ND	ND/ND	−/ND	−/−	+/ND	ND/ND	ND/ND	ND/ND	ND/ND	ND/ND	ND/ND	V
*P. aeruginosa* -11 VIM	ND/ND	ND/ND	ND/ND	ND/ND	ND/ND	ND/ND	ND/ND	ND/ND	ND/ND	ND/ND	−/−	−/−	−/−	−/−	+/+	+/+	V

Briefly, colonies are assessed for their presence, color (on chromogenic plates only), staining properties and shape, ID using MALDI-ToF-MS, susceptibility (Radian^®^, E-tests, Sensititre™), and resistance gene content by loop amplification (MDx). Results are then reported as correct in green (presence of the expected inoculated strain depicted by a V) or false in red (e.g., contaminant, lack of growth of the target species or any unexpected result, depicted by an X). Gray boxes refer to non-monitored determinations. GNR: Gram-negative rods, ND: not determined, V: valid, X: invalid; VIM: carbapenemase-resistance gene VIM (Verona Integron-encoded metallo-β-lactamase), +: expected colony growth and morphology and/or expected colony color, −: expected absence of growth and expected absence of colony color.

Since 2024, our analytic workflow has been assisted by PhenoMATRIX^®^, an artificial intelligence-powered system, for the screening of negative cultures (e.g., MRSA or urine; Cherkaoui et al., 2024; Dauwalder et al., 2021) and validation of the bacterial strains that are detected on selective agar plates (group B streptococci; Baker et al., 2020; Cherkaoui et al., 2025a). PhenoMATRIX^®^ algorithms were also assessed by our integrated IQC panel (seeding protocols 3 and 4, **Table 2**).

AST determination by automated disc diffusion was evaluated weekly, while microdilution assays by Sensititre™ and E-tests assays were evaluated monthly and interpreted according to EUCAST v15 recommendations (https://www.eucast.org/ast_of_bacteria/quality_control).

Finally, molecular biology was performed on selected strains carrying ESBL (CTXM-1 group), Oxa48, VIM, and Oxa23 resistance determinants (**Figure 1b**; **Table 1**).

Results were collected monthly following the procedures illustrated for the two illustration strains shown in **Figure 1** and **Table 3**. Expected results (row tests) that were confirmed were flagged by a green tick mark. Any error was reported by a red flag. Expected result outputs are detailed in **Table 3**. Seeding is evaluated as correct if growth, staining, and identification are reported as expected. The staining is reported as correct if the expected color and shape are observed. For example, *E. coli* shall be reported as a Gram-negative rod abbreviated GNR in **Table 3**. Growth on each medium is assessed based on the expected colony morphology on the agar plate, the presence of a single type of morphology, and the correct colony color whenever the latter is relevant (e.g., on a chromogenic agar). Thus, for each plate and each bacterium, a specific pattern of expected growth and color is defined with a “+” for the correct growth and shape or color, “−” for no growth or absence/incorrect color, and “ND” when the parameter is not (or cannot be) evaluated. For example, *P. aeruginosa* -11 VIM shall not grow on MRSA-ID agar plate; therefore, the expected results are −/−; for *E. coli* ATCC 25922, growth on GS agar is expected and is represented by a “+”, but GS is not a chromogenic plate; thus, no color can be monitored, and this is represented as “ND.” If growth and color on each growth medium are correct, the final evaluation will be noted as “V” for valid. Identification of bacteria is performed using MALDI-ToF-MS. We accept as valid an ID score >2.00 and only a single bacterial identification appearing in the identification list. AI-assisted plate reading is, like seeding, evaluated indirectly during plate reading by the technologists. PhenoMATRIX^®^ will automatically validate negative plates for urine culture and for MRSA and *S. agalactiae* screening. Positive plates are sent for human reading. Negative sample e-swabs 2-3-4 (**Table 1**) must be reported negative for MRSA, urine culture, and *S. agalactiae* screening with negative results sent by PhenoMATRIX^®^ to our LIS and automatically validated. Positive cultures must be worked up by a technologist. An error with PhenoMATRIX^®^ would result in a missing negative result in our LIS. The range for disc diffusion and MIC AST must be strictly within the defined range to be accepted. For example, for *P. aeruginosa* -11 VIM, the target MIC for colistin is 1 µg/mL, and its accepted range is 0.5–2 µg/mL; for *E. coli* ATCC 25922, the target value is 26 mm, and its accepted range is 23–29 mm according to EUCAST. For resistance gene detection, the result is accepted if the expected gene is detected and all the other targets remain negative.

During 6 months, we ran 6 cycles of monthly integrated IQC panel and 24 cycles of weekly AST testing using disc diffusion. A total of 96 chromogenic and non-chromogenic plates were used for each monthly cycle of the integrated IQC panel. In addition, 7 staining procedures, 18 MALDI spots, and 4 resistance gene detection panels were performed. For AST testing, we used monthly 221 antibiotic discs, 8 E-tests strips, and 3 Sensititre™ plates (encompassing a total of 41 antibiotics tested, including 14 per MDR plate and 13 for the anaerobic plate). Over 6 months of monitoring, we recorded 100% adequate IQC results regarding staining quality, culture growth, colony color or morphology, contamination, identification, automated disc diffusion, and molecular resistance gene detection (**Supplementary Table 1**). However, we detected one issue with a MIC determination (**Supplementary Table 2**), representing a total failure rate of 0.04% over the 6-month period or 0.06% when considering only AST. We identified one misreading for imipenem using the Sensititre™ assay for anaerobic bacteria (*B. fragilis* ATCC25285). The first manual reading reported a MIC of 0.25 mg/L for imipenem, which was above the upper MIC range published by EUCAST (i.e., 0.125 mg/L). This is a well-known reading error, previously described by the manufacturer, warning that “there may be a gradual fading of growth over 2 to 3 wells.” Nevertheless, as stated in the manufacturer’s package insert, “the end points should be taken as the first well that inhibits visible growth” [Sensititre® Anaerobe MIC SUSCEPTIBILITY PLATES (*B. fragilis* group) insert]. The correct value was 0.125mg/L. Correction was therefore made, and collaborators retrained for appropriate Sensititre™ plate reading, as suggested by the manufacturer’s package insert.

## Discussion

The composition of the proposed IQC panel may vary between laboratories, depending on the size of the laboratory’s operations, the culture media used, and the equipment. We set up and validated an IQC panel on a COPAN’s WASPLab automation system coupled with independent instruments such as MALDI-ToF-MS from Bruker and an automated stainer from bioMérieux. Each instrument has inherent limitations and is tested and maintained separately within our independent quality control system. Reference strains such as *E. coli* ATCC 25922 or *S. aureus* ATCC 29213 were selected for their clinical relevance in a hospital setting, and specific ATCC strains for the availability of EUCAST recommendations for AST QC. Reference strains like *Enterococcus faecium* VRE ATCC 700221, *E. coli* -9 BLSE, or carbapenemase gene-carrying *P. aeruginosa* -11 VIM and *A. baumanii* -12 Oxa23 were selected to control our “infection prevention and control workflow” dedicated to multidrug-resistant bacteria screening upon hospital admission. Other laboratories would have to select reference strains and integrate IQC tests based on their performed analyses and the instruments used in their laboratories. These changes cannot be evaluated in this study, nor could we assess another automated culture system; however, this integrated IQC panel can be used as a template and be adapted to fit local specifications and requirements.

As requested in all diagnostic laboratories, we perform an independent IQC for every analysis and instrument. The risk of misidentifying device failure is limited due to the independent IQC performed in the laboratory, such as the weekly BTS testing for MALDI-ToF-MS, which confirms that individual analysis remains reliable. For Eazyplex^®^, every positive sample is sent to a reference center, thus confirming the accuracy of the method. IQC for disc diffusion AST is performed independently every week and included in the integrated IQC panel once a month. In addition, external quality assessment (EQA) is performed for each analysis, two to four times per year, to monitor equipment and analysis performance as compared against other laboratories. However, no controls are currently used to regularly test the performance of all the systems integrated for automated culture analyses. Performing this additional integrated IQC once a month was therefore selected as an optimal balance between workload and drift detection capability.

Every parameter measured with the integrated IQC panel must be valid to pass. If one parameter is incorrect, an investigation must be conducted to determine whether the error is of human or technical nature, and the error must be defined as well as the way to resolve it. For example, if a MALDI-ToF-MS score is below 2.0, we will first control the value. If the value is between 1.8 and 2.0, we will repeat the identification test. If the score is below 1.8 and the strain has been on the plate for more than 48 h (e.g., over a weekend), the strain must be re-isolated and tested again for identification. If the strain has been on the plate for less than 24 h with a score below 1.8, the MALDI-ToF-MS malfunction should be investigated. The issue is often related to laser calibration and can be rapidly resolved through remote maintenance. If the strain is misidentified, it is also highly likely that the technologist identified an incorrect morphology on the agar plate. The first action is to control plate sterility at *t* = 0. All automated systems take pictures immediately after seeding, allowing users to check for plate sterility. If a contamination is identified on the plate, the lot number is recorded and the remaining plates with the same lot number are inspected. Isolated contamination happens, and in such a case, the specific IQC number must be performed again. If multiple plates from the same shipping box are contaminated, a report is created and communicated to the manufacturer, and all plates with that same lot number are discarded. IQC must then be repeated with the new lot. Misidentification as well as incorrect morphology might also result from human error during the preparation stage, when strains are introduced on e-swabs and barcodes are generated. Either an incorrect barcode or an incorrect strain may have been used. A new IQC sample test tube must be created and tested alongside the original sample related to the identification error. A similar procedure should be applied if an incorrect staining is recorded. Invalid AST results must be investigated separately. When the measured diameter is 1 mm (or one MIC value dilution) outside of the accepted range, reviewing the antibiotic susceptibility reading often allows correction of the error, if related to misreading by the technologist. A simple reminder on how to read disc diffusion inhibition zones (or MIC by E-tests or serial dilutions) is required. However, if the measured value is significantly outside the expected range, several actions must be undertaken. First, strain identification must be performed within the expected inhibition zone to rule out possible contamination with another strain. If another strain is present, antibiogram agar plates (disc diffusion and E-tests) must be controlled for sterility (a picture at *t* = 0 is also taken after seeding and disc diffusion placement on the plate before incubation). For Sensititre AST, identification can be performed from the purity control plate. If identification is incorrect, AST must be repeated and Sensititre AST performed that same day should be controlled to prevent a sample mix-up event. If identification is correct, the antibiotic may be degraded, and AST must be repeated. If the error persists, a different lot number must be used, and all AST performed with the problematic antibiotic shall be reevaluated. A report is also generated and communicated to the manufacturer, and antibiotics from the same lot number must be discarded. In addition, antibiograms with the defective antibiotic shall be manually reviewed.

Implementing an IQC panel implies both financial and time investments. The time dedicated to setting up and validating the IQC workflow, as well as to preparing strain aliquots, which can be used for several years, was not included in the final cost estimation. Reported costs included reagents and consumables amounting to CHF 8,343 and technologist (dedicated and routine) and staff working time amounting to CHF 9,543.60 (**Table 4**). Integrated IQC offers the assurance of quality results and comes with a marginal additional cost. However, this cost must be added to the already existing IQC, EQA, and to the application of the new IVD-R regulation (Coste et al., 2023).

**Table 4:** Integrated IQC cost evaluation.

2024	Routine load (*n*/month)	IQC load (*n*/month)	IQC load (%)	Cost per unit (CHF)	Cost per month (CHF)	Cost per year (CHF)	% of total cost
Non-chromogenic agar plates	17,241	48	0.3%	1.18	56.64	679.7	
Chromogenic agar plates	8,083	48	0.6%	1.93	92.64	1,111.7	
Staining procedures	3,189	7	0.2%	0.91	6.36	76.3	
MALDI-ToF-MS spots	3,450	18	0.5%	0.31	5.58	67.0	
Antibiotic discs	26,790	221	0.8%	0.33	72.93	875.2	
E-tests	302	8	2.6%	6.4	51.20	614.4	
Sensititre tests	42	3	7.1%	36.4	109.20	1,310.9	
PCR panels	41	4	9.8%	75.17	300.66	3,607.9	
**Total cost for reagents and consumable**					**695.2**	**8,343.0**	46.6%
Dedicated technologist work time (min)	147,900	190	0.1%	0.9	171	2,052.0	
Routine technologist work time (min)		599	0.4%	0.9	539.10	6,469.2	
Staff time (min)	12,000	60	0.5%	1.42	85.20	1,022.4	
**Total cost for salaries**					**795.3**	**9,543.6**	**53.4%**
**Total costs for Reagents, consumable and salaries (CHF)**					**1,490.5**	**17,886.6**	

Integrated IQC workload, unit costs, and annual costs associated with the different categories of consumables and personnel time for the year 2024. The table evaluates the monthly IQC workload to the routine workload, as well as the estimated monthly and annual integrated IQC costs. Costs are grouped into two main categories: reagents and consumables, and personnel time. For the purpose of comparison, personnel time was reported in minutes and was compared to the overall routine load. In addition, the technologist time was separated between a dedicated technologist, in charge of reference strains handling, IQC preparation [e-swabs (COPAN, Brescia, Italy) for seeding and informatics workload] and results collection, and routine technologists, involved in the IQC analysis process included in the routine workflow. Bold text and values refer to total costs and percentage either for reagents and consumables, salaries or both.

This integrated IQC panel allows continuous monitoring of all the culture-based processes using TLA. Although we tried to simplify the process as much as possible, some critical time-consuming steps remain to be optimized. Firstly, test prescriptions were manually created, one by one, in the LIS. Software development allowing automatic prescriptions could significantly decrease the time required for this step and the risk of transcription errors. Secondly, results were manually collected, tabulated, and analyzed in a spreadsheet. Implementing specialized IQC software to automatically create prescription and collect, analyze, report, and archive data may help in improving the efficiency and traceability of our IQC results.

Implementation of this IQC provides an accurate surveillance of processes on TLA, ensuring the quality of the results, by addressing current methodological and operational challenges of automated microbiology laboratories.

## Data Availability

The original contributions presented in the study are included in the article/**Supplementary Material**. Further inquiries can be directed to the corresponding author.
