# Supplementary material for: Total laboratory automation-based monitoring processes: setup and validation of an integrated internal quality control panel
Source: Front Cell Infect Microbiol. 2026 May 6;16:1771552. doi: 10.3389/fcimb.2026.1771552 (PMC13188209; doi:10.3389/fcimb.2026.1771552)
Supplement: Supplementary file 1 [file Table1.docx]

**Supplementary files**

**Supplementary table 1**

| ICQ strains | Dates Tests | 25.03.2025 | 14.04.2025 | 16.05.2025 | 16.06.2025 | 28.07.2025 | 22.08.2025 |
| --- | --- | --- | --- | --- | --- | --- | --- |
| *Staphylococcus aureus* ATCC 29213 | COS POS | v | v | v | v | v | v |
|  | CNA POS | v | v | v | v | v | v |
|  | MCK NEG | v | v | v | v | v | v |
|  | PVX POS | v | v | v | v | v | v |
|  | BHI-GC NEG | v | v | v | v | v | v |
|  | CAC NEG | v | v | v | v | v | v |
|  | BHI POS | v | v | v | v | v | v |
|  | MALDI | v | v | v | v | v | v |
|  | Staining | v | v | v | v | v | v |
|  | AST (Weekly disc diffusion + MIC Supplementary Table 2) | v | v | v | v | v | v |
| *Pseudomonas aeruginosa* ATCC 10145 | CPSE POS | v | v | v | v | v | v |
|  | COS POS | v | v | v | v | v | v |
|  | CNA NEG | v | v | v | v | v | v |
|  | MCK POS L- | v | v | v | v | v | v |
|  | PVX POS | v | v | v | v | v | v |
|  | BHI-GC NEG | v | v | v | v | v | v |
|  | CAC NEG | v | v | v | v | v | v |
|  | BHI POS | v | v | v | v | v | v |
|  | MALDI | v | v | v | v | v | v |
|  | Staining | v | v | v | v | v | v |
|  | AST (MIC Supplementary Table 2) | v | v | v | v | v | v |
| *Escherichia coli* ATCC 25922 | CPSE POS | v | v | v | v | v | v |
|  | COS POS | v | v | v | v | v | v |
|  | CNA NEG | v | v | v | v | v | v |
|  | MCK POS L+ | v | v | v | v | v | v |
|  | PVX POS | v | v | v | v | v | v |
|  | BHI-GC NEG | v | v | v | v | v | v |
|  | CAC NEG | v | v | v | v | v | v |
|  | BHI POS | v | v | v | v | v | v |
|  | MALDI | v | v | v | v | v | v |
|  | MDR | v | v | v | v | v | v |
|  | Staining | v | v | v | v | v | v |
|  | AST (Weekly disc diffusion + MIC Supplementary Table 2) | v | v | v | v | v | v |
| *Enterococcus faecalis* ATCC 29212 | CPSE POS | v | v | v | v | v | v |
|  | COS POS | v | v | v | v | v | v |
|  | CNA POS | v | v | v | v | v | v |
|  | MCK NEG | v | v | v | v | v | v |
|  | PVX POS | v | v | v | v | v | v |
|  | BHI-GC NEG | v | v | v | v | v | v |
|  | CAC NEG | v | v | v | v | v | v |
|  | BHI POS | v | v | v | v | v | v |
|  | MALDI | v | v | v | v | v | v |
|  | AST (MIC Supplementary Table 2) | v | v | v | v | v | v |
| *Haemophilus influenzae* ATCC 10211 | COS NEG | v | v | v | v | v | v |
|  | CNA NEG | v | v | v | v | v | v |
|  | MCK NEG | v | v | v | v | v | v |
|  | PVX POS | v | v | v | v | v | v |
|  | BHI-GC NEG | v | v | v | v | v | v |
|  | CAC NEG | v | v | v | v | v | v |
|  | BHI POS | v | v | v | v | v | v |
|  | MALDI | v | v | v | v | v | v |
|  | AST (MIC Supplementary Table 2) | v | v | v | v | v | v |
| *Streptococcus agalactiae* ATCC 12386 | STRB POS | v | v | v | v | v | v |
|  | MALDI | v | v | v | v | v | v |
| *Staphylococcus aureus* MRSA USA300 JE2 | SAIDE POS | v | v | v | v | v | v |
|  | MRSA POS | v | v | v | v | v | v |
|  | VRE NEG | v | v | v | v | v | v |
|  | Acin NEG | v | v | v | v | v | v |
|  | ESBL NEG | v | v | v | v | v | v |
|  | OXA NEG | v | v | v | v | v | v |
|  | MALDI | v | v | v | v | v | v |
| *Enterococcus faecium* VRE ATCC 700221 | SAIDE NEG | v | v | v | v | v | v |
|  | MRSA NEG | v | v | v | v | v | v |
|  | VRE POS | v | v | v | v | v | v |
|  | Acin NEG | v | v | v | v | v | v |
|  | ESBL NEG | v | v | v | v | v | v |
|  | OXA NEG | v | v | v | v | v | v |
|  | MALDI | v | v | v | v | v | v |
| *Escherichia coli* -9 ESBL | SAIDE NEG | v | v | v | v | v | v |
|  | MRSA NEG | v | v | v | v | v | v |
|  | VRE NEG | v | v | v | v | v | v |
|  | Acin NEG | v | v | v | v | v | v |
|  | ESBL POS | v | v | v | v | v | v |
|  | OXA NEG | v | v | v | v | v | v |
|  | MALDI | v | v | v | v | v | v |
|  | amplex CTXM POS | v | v | v | v | v | v |
| *Klebsiella pneumoniae -10* Oxa48 | SAIDE POS | v | v | v | v | v | v |
|  | MRSA POS | v | v | v | v | v | v |
|  | VRE NEG | v | v | v | v | v | v |
|  | Acin NEG | v | v | v | v | v | v |
|  | ESBL NEG | v | v | v | v | v | v |
|  | OXA POS | v | v | v | v | v | v |
|  | MALDI | v | v | v | v | v | v |
|  | amplex CTXM NEG | v | v | v | v | v | v |
|  | amplex OXA POS | v | v | v | v | v | v |
| *Pseudomonas aeruginosa -11* VIM | SAIDE NEG | v | v | v | v | v | v |
|  | MRSA NEG | v | v | v | v | v | v |
|  | VRE NEG | v | v | v | v | v | v |
|  | Acin NEG | v | v | v | v | v | v |
|  | ESBL POS | v | v | v | v | v | v |
|  | OXA POS | v | v | v | v | v | v |
|  | MALDI | v | v | v | v | v | v |
|  | amplex VIM POS | v | v | v | v | v | v |
|  | AST (MIC Supplementary Table 2) | v | v | v | v | v | v |
| *Acinetobacter* *baumanii* -12 Oxa23 | SAIDE NEG | v | v | v | v | v | v |
|  | MRSA NEG | v | v | v | v | v | v |
|  | VRE NEG | v | v | v | v | v | v |
|  | Acin POS | v | v | v | v | v | v |
|  | ESBL NEG | v | v | v | v | v | v |
|  | OXA POS | v | v | v | v | v | v |
|  | MALDI | v | v | v | v | v | v |
|  | amplex OXA23 POS | v | v | v | v | v | v |
| *Pichia kudriavzevii*  (previously: *Candida krusei*) ATCC 6258 | COS POS | v | v | v | v | v | v |
|  | CNA POS | v | v | v | v | v | v |
|  | MCK NEG | v | v | v | v | v | v |
|  | PVX POS | v | v | v | v | v | v |
|  | BHI-GC POS | v | v | v | v | v | v |
|  | CAC POS | v | v | v | v | v | v |
|  | BHI POS | v | v | v | v | v | v |
|  | MALDI | v | v | v | v | v | v |
|  | Staining | v | v | v | v | v | v |
| *Bacteroides fragilis* ATCC 25285 | COS NEG | v | v | v | v | v | v |
|  | MCK NEG | v | v | v | v | v | v |
|  | CNA NEG | v | v | v | v | v | v |
|  | PVX NEG | v | v | v | v | v | v |
|  | CDC POS | v | v | v | v | v | v |
|  | AST (MIC Supplementary Table 2) | v | v | v | v | v | v |
| e-swab 1 (negative control) | COS NEG | v | v | v | v | v | v |
|  | CNA NEG | v | v | v | v | v | v |
|  | MCK NEG | v | v | v | v | v | v |
|  | PVX NEG | v | v | v | v | v | v |
|  | BHI-GC NEG | v | v | v | v | v | v |
|  | CAC NEG | v | v | v | v | v | v |
|  | BHI NEG | v | v | v | v | v | v |
| e-swab 2 (negative control) | SAIDE NEG | v | v | v | v | v | v |
|  | MRSA NEG | v | v | v | v | v | v |
|  | VRE NEG | v | v | v | v | v | v |
|  | Acin NEG | v | v | v | v | v | v |
|  | ESBL NEG | v | v | v | v | v | v |
|  | OXA NEG | v | v | v | v | v | v |
| e-swab 3 (negative control) | CPSE NEG | v | v | v | v | v | v |
| e-swab 4 (negative control) | STRB NEG | v | v | v | v | v | v |

**Supplementary table 1: Results output.** Final evaluation for each parameter is computed for each reference strain. Every unexpected result is flagged in red and has to be investigated
