# Supplementary material for: Total laboratory automation-based monitoring processes: setup and validation of an integrated internal quality control panel
Source: Front Cell Infect Microbiol. 2026 May 6;16:1771552. doi: 10.3389/fcimb.2026.1771552 (PMC13188209; doi:10.3389/fcimb.2026.1771552)
Supplement: Supplementary file 2 [file Table2.docx]

**Supplementary table 2**

| ICQ date | 25.03.2025 | 14.04.2025 | 16.05.2025 | 16.06.2025 | 28.07.2025 | 22.08.2025 | 23.09.2025 |
| --- | --- | --- | --- | --- | --- | --- | --- |
| ***E. coli* 25922 (target / range)** |  |  |  |  |  |  |  |
| Amikacine (1-2 / 0.5-4) | <= 2 | <= 2 | <= 2 | <= 2 | <= 2 | <= 2 | <= 2 |
| Aztreonam (0.125-0.25 / 0.06-0.5) | <= 1 | <= 1 | <= 1 | <= 1 | <= 1 | <= 1 | <= 1 |
| Cefepime (0.03-0.06 / 0.016-0.125) | <= 1 | <= 1 | <= 1 | <= 1 | <= 1 | <= 1 | <= 1 |
| Ceftazidime/Avibactam (0.125-0.25 / 0.06-0.5) | <= 0.25 | <= 0.25 | <= 0.25 | <= 0.25 | <= 0.25 | <= 0.25 | <= 0.25 |
| Ceftolozane/Tazobactam 4 (0.25 / 0.125-0.5) | <= 0.25 | <= 0.25 | <= 0.25 | <= 0.25 | <= 0.25 | 0.25 | <= 0.25 |
| Colistine (0.5 / 0.25-1) | <= 0.5 | <= 0.5 | <= 0.5 | <= 0.5 | <= 0.5 | <= 0.5 | <= 0.5 |
| Eravacycline (0.06 / 0.03-0.125) | 0.12 | 0.12 | 0.12 | 0.12 | 0.12 | 0.12 | 0.12 |
| Fosfomycine + G6P (1 / 0.5-2) | <= 16 | <= 16 | <= 16 | <= 16 | <= 16 | <= 16 | <= 16 |
| Imipenem/Relebactam (0.125-0.25 / 0.06-0.5) | 0.12 | 0.5 | 0.12 | 0.12 | 0.25 | 0.12 | 0.12 |
| Imipenem (0.125-0.25 / 0.06-0.5) | <= 1 | <= 1 | <= 1 | <= 1 | <= 1 | <= 1 | <= 1 |
| Meropenem/Vaborbactam (0.016-0.03 / 0.008-0.06) | <= 0.06 | <= 0.06 | <= 0.06 | <= 0.06 | <= 0.06 | <= 0.06 | <= 0.06 |
| Meropenem ((0.016-0.03 / 0.008-0.06) | <= 0.12 | <= 0.12 | <= 0.12 | <= 0.12 | <= 0.12 | <= 0.12 | <= 0.12 |
| Piperacillin/Tazobactam (2-4 / 1-8)) | <= 4 | <= 4 | <= 4 | <= 4 | <= 4 | <= 4 | <= 4 |
| Tigecycline (0.06-0.125 / 0.03-0.25) | <= 0.5 | <= 0.5 | <= 0.5 | <= 0.5 | <= 0.5 | <= 0.5 | <= 0.5 |
| Tobramycin (0.5 / 0.25-1) | 1 | <= 0.5 | <= 0.5 | <= 0.5 | <= 0.5 | <= 0.5 | <= 0.5 |
| Cefiderocol (0.125-0.25 / 0.06-0.5) | 0.25 | 0.5 | 0.5 | 0.5 | 0.5 | 0.25 | 0.25 |
|  |  |  |  |  |  |  |  |
|  |  |  |  |  |  |  |  |
| ICQ date | 25.03.2025 | 14.04.2025 | 16.05.2025 | 16.06.2025 | 28.07.2025 | 22.08.2025 | 23.09.2025 |
| ***P. aeruginosa -11* VIM (target / range)** |  |  |  |  |  |  |  |
| Amikacine (16 / 8-32)* | 16 | 16 | 32 | 16 | 32 | 16 | 16 |
| Aztreonam (8 / 4-16)* | 4 | 8 | 4 | 4 | 8 | 4 | 4 |
| Cefepime (16 / 8->16)* | 16 | 16 | > 16 | 16 | > 16 | > 16 | > 16 |
| Ceftazidime/Avibactam (16 / 8->16)* | 16 | 16 | > 16 | 16 | > 16 | > 16 | > 16 |
| Ceftolozane/Tazobactam (8 / 4->8)* | 8 | 8 | >8 | 8 | >8 | >8 | >8 |
| Colistine (1 / 0.5-2)* | 1 | 1 | 1 | 1 | 1 | 1 | 1 |
| Eravacycline (0.5 / 0.25->0.5)* | 0.5 | 0.5 | > 0.5 | 0.5 | > 0.5 | > 0.5 | > 0.5 |
| Fosfomycine + G6P (32 / 16-64)* |  | 64 | 32 | 32 | 32 | 32 | 32 |
| Imipenem/Relebactam (8 / 4->8)* | 8 | 8 | > 8 | 8 | > 8 | > 8 | > 8 |
| Imipenem (8 / 4->8)* | 8 | 8 | > 8 | 8 | > 8 | > 8 | > 8 |
| Meropenem/Vaborbactam (16 / 8->16)* | 16 | 16 | > 16 | 16 | > 16 | > 16 | > 16 |
| Meropenem (16 / 8->16)* | 16 | 16 | > 16 | 16 | > 16 | > 16 | > 16 |
| Piperacillin/Tazobactam (32 / 16->32)* | 32 | 32 | > 32 | 32 | > 32 | > 32 | > 32 |
| Tigecycline (1 / 0.5->1)* | 1 | 1 | > 1 | 1 | > 1 | > 1 | > 1 |
| Tobramycin (4 / 2->4)* | 4 | 4 | > 4 | 4 | > 4 | > 4 | > 4 |
| Cefiderocol (0.25-0.5 / 0.125-1)* | 0.5 | 0.125 | 0.25 | 0.125 | 1 | 1 | 1 |
|  |  |  |  |  |  |  |  |
|  |  |  |  |  |  |  |  |
| ICQ date | 25.03.2025 | 22.04.2025 | 16.05.2025 | 16.06.2025 | 28.07.2025 | 22.08.2025 | 23.09.2025 |
| ***B. fragilis* 25285 (target / range)** |  |  |  |  |  |  |  |
| Amoxicillin/Calvulanic acid (0.125 / 0.06-0.25) | <= 0.25 | <= 0.25 | <= 0.25 | <= 0.25 | <= 0.25 | <= 0.25 | <= 0.25 |
| Amoxicillin (8 / 4-16)* | 8 | 4 | 4 | 8 | 8 | 8 | 8 |
| Cefoxitin (1 / 0.5-2)* | 1 | 1 | 2 | 1 | 1 | 1 | 1 |
| Chloramphenicol (4 / 2-8)* | 4 | 4 | 4 | 4 | 4 | 4 | 4 |
| Clindamycin (1 / 0.5-2) | <= 0.5 | <= 0.5 | <= 0.5 | <= 0.5 | <= 0.5 | <= 0.5 | <= 0.5 |
| Erythromycin (1 / 0.5-2)* | <= 1 | <= 1 | <= 1 | <= 1 | <= 1 | <= 1 | <= 1 |
| Imipenem (0.06 / 0.03-0.125) | <= 0.06 | 0.125 | <= 0.06 | <= 0.06 | 0.125^$^ | 0.125 | <=0.06 |
| Metronidazole (0.5 / 0.25-1) | 0.5 | 1 | <=0.5 | <=0.5 | 1 | 1 | 1 |
| Moxifloxacine (0.25 / 0.125-0.5)* | 0.25 | 0.25 | <=0.12 | 0.25 | <=0.12 | 0.25 | 0.25 |
| Penicillin (8 / 4-16)* | 8 | 8 | 8 | 8 | 8 | 8 | 8 |
| Metronidazole (0.5 / 0.25-1) | <= 16 | <= 16 | <= 16 | <= 16 | <= 16 | <= 16 | <= 16 |
| Piperacillin (<=16)* | <= 16 | <= 16 | <= 16 | <= 16 | <= 16 | <= 16 | <= 16 |
| Tetracycline (2 / 1-4)* | <= 2 | <= 2 | <= 2 | <= 2 | <= 2 | <= 2 | <= 2 |
| Vancomycin (8 / 4-16)* | 8 | 8 | 8 | 8 | 8 | 4 | 4 |
|  |  |  |  |  |  |  |  |
|  |  |  |  |  |  |  |  |
| ICQ date | 25.03.2025 | 14.04.2025 | 16.05.2025 | 16.06.2025 | 28.07.2025 | 22.08.2025 | 23.09.2025 |
| ***E. coli* 25922 E-test (target / range)** |  |  |  |  |  |  |  |
| Amoxicillin/Clavulanic acid (4 / 2-8) | 8 | 8 | 6 | 8 | 8 | 6 | 6 |
| Ciprofloxacin (0.008 / 0.004-0.016) | 0.012 | 0.016 | 0.012 | 0.012 | 0.012 | 0.012 | 0.012 |
| Meropenem (0.016-0.03 / 0.008-0.06) | 0.064 | 0.032 | 0.023 | 0.032 | 0.023 | 0.032 | 0.032 |
|  |  |  |  |  |  |  |  |
|  |  |  |  |  |  |  |  |
| ***H. influenzae* 10211 E-test (target / range)** |  |  |  |  |  |  |  |
| Ampicillin (0.19 / 0.06-0.38)* | 0.19 | 0.19 | 0.38 | 0.19 | 0.125 | 0.125 | 0.125 |
| Ceftriaxone (0.002 / 0.001-0.004)* | 0.002 | 0.002 | 0.002 | 0.003 | 0.002 | 0.002 | 0.002 |
|  |  |  |  |  |  |  |  |
|  |  |  |  |  |  |  |  |
| ***S. aureus* 29213 E-test (target / range)** |  |  |  |  |  |  |  |
| Daptomycin (0.25-0.5 / 0.125-1) | 0.5 | 0.38 | 0.38 | 0.38 | 0.38 | 0.75 | 0.75 |
| Teicoplanin (0.5 / 0.25-1) | 1 | 1 | 1 | 1 | 0.38 | 1 | 1 |
| Vancomycin (1 / 0.5-2) | 2 | 2 | 1.5 | 2 | 2 | 2 | 2 |

**Supplementary table 2: MIC results output** for selected reference strains. * Target and range set by the lab (cf M&M); $: Sensititre MIC reading error.
